# Body Mass Index and Risk of Prostate Volume, International Prostate Symptom Score, Maximum Urinary Flow Rate, and Post-Void Residual in Benign Prostatic Hyperplasia Patients

**DOI:** 10.1177/1557988319870382

**Published:** 2019-08-19

**Authors:** Bing-Hui Li, Tong Deng, Qiao Huang, Hao Zi, Hong Weng, Xian-Tao Zeng

**Affiliations:** 1 Department of Urology, Zhongnan Hospital of Wuhan University, Wuhan, China; 2 Center for Evidence-Based and Translational Medicine, Zhongnan Hospital of Wuhan University, Wuhan, China; 3 Center for Evidence-Based Medicine, Institute of Evidence-Based Medicine and Knowledge Translation, Henan University, Kaifeng, China

**Keywords:** body mass index, benign prostatic hyperplasia, prostate volume, international prostate symptom score, maximum urinary flow rate, post-void residual

## Abstract

The objective of this study was to evaluate association between body mass index (BMI) and prostate volume (PV), international prostate symptom scores (IPSS), maximum urinary flow rate (*Q*_max_), and post-void residual (PVR) of Chinese benign prostatic hyperplasia (BPH) patients. All newly diagnosed BPH patients between September 2016 and August 2018 were selected and 788 patients were included. According to BMI, the patients were categorized into four groups, while according to PV, IPSS, *Q*_max_, and PVR, they were categorized into two groups based on clinical significant cutoffs. Univariable and multivariable logistic regressions and a restricted cubic spline (RCS) were applied to explore the relationship of BMI with categorical PV, IPSS, *Q*_max_, and PVR. Compared with normal BMI, obesity presented significant association with increased risk of larger PV (>80 ml) in both unadjusted and adjusted models (unadjusted odds ratio [OR] = 1.772, 95% CI [1.201, 2.614], *p* = .004; adjusted OR = 1.912, 95% CI [1.212, 3.017], *p* = .005); however, underweight or overweight did not present a significant connection with such risk. No significant effect was identified for BMI on IPSS, *Q*_max_, or PVR in either unadjusted or adjusted model. Nonlinear test including BMI using RCS and adjusting for confounders showed no significance (*p* > .05); however, a significant linear relationship was ascertained between BMI and the risk of larger PV (*p* < .001). In conclusion, there was a significant linear association between BMI and the risk of larger PV in BPH patients. Hence, this suggests urologists should consider both BMI and PV when providing surgical treatment for BPH patients.

Obesity has become an issue of worldwide concern and its prevalence is increasing at a faster rate than ever before (Parikh et al., 2007). A number of epidemiological studies demonstrate that obesity maybe a risk factor for multiple physical and mental problems, such as cancers (Colditz & Peterson, 2018), cardiovascular diseases (Parto & Lavie, 2017), Alzheimer’s disease (Alford, Patel, Perakakis, & Mantzoros, 2018), sleep (Ogilvie & Patel, 2017), and benign prostatic hyperplasia (BPH; Jung et al., 2016). BPH is one of the most common diseases in aged men, which is defined histologically by smooth muscle and epithelial cell proliferation in prostate transition zone, resulting in nonmalignant prostate enlargement (Priest, Garzotto, & Kaufman, 2012; Roehrborn, 2008; Santos Dias, 2012). BPH not only causes discomfort for the person but it also decreases the quality of life (QoL; Larsen & Post, 2013), and relevant researches identify that one in three men aged over 80 years needs treatment to relieve BPH-induced symptoms (Luo et al., 2002; Pinheiro & Martins Pisco, 2012; Priest et al., 2012; Santos Dias, 2012).

Although BPH is considered to be associated with sex steroid hormone levels and has a genetic predisposition (Fang et al., 2017; Oelke et al., 2013; Su, Zeng, Fang, Liu, & Wang, 2017; Zeng et al., 2014), its exact etiology and pathogenesis remain unclear. Clinical characteristics of prostate in BPH cases can be determined by prostate volume (PV), International Prostate Symptom Score (IPSS), and maximum urinary flow rate (*Q*_max_). When performing surgical treatment for BPH, final efficacy can be influenced by clinical characteristics of BPH. For instance, according to the European Association of Urology (EAU) Guidelines, monopolar transurethral resection of the prostate (TURP) is a preferred management option for prostates with a volume between 30 and 80 cc (Oelke et al., 2013). It is very important to identify risk factors for negative PV (more than 80 cc), IPSS, and *Q*_max_. Two large cohort studies have indicated obesity is related to the risk of symptomatic BPH (Giovannucci et al., 1994; Kristal et al., 2007), but the influence of body mass index (BMI) on PV, IPSS, and *Q*_max_ have not yet been conclusively assessed. Besides, post-void residual (PVR) urine is an important index evaluating conditions for any patient presenting with lower urinary tract symptoms (LUTS), and in 2010, a study indicated that BMI and genital hiatus length might play an important role in post-void dribbling, especially in postmenopausal women (Ablove, 2010); however, the association between BMI and PVR in BPH patients remains blurred. Therefore, this study was performed to explore the relationship of BMI with PV, IPSS, *Q*_max_, and PVR in Chinese BPH patients.

## Materials and Methods

### Design and Ethics Statement

This cross-sectional study was conducted and reported according to the Strengthening the Reporting of Observational studies in Epidemiology (STROBE) statement (Vandenbroucke et al., 2014). Baseline data for this study were obtained from the Bladder Cancer and Benign Prostatic Hyperplasia Study in Chinese Population (BPSC; Zeng, Liu et al., 2018; Zeng, Weng, Jin et al., 2018; Zeng, Weng, Xiong et al., 2018), which was a prospective study to estimate interventional effect on and risk factors for bladder cancer and BPH. All participants were Chinese people and had signed an informed consent before enrollment. This study had been reviewed and approved by the Committee for Ethical Affairs of the Zhongnan Hospital of Wuhan University at Wuhan City, Hubei Province.

### Study Population and Data

Participants were recruited between September 2016 and August 2018 and were diagnosed through urological examination. They all underwent strict examinations on their medical histories and physical statuses. Patients would be excluded if they met any one of the following conditions: (a) not receiving ultrasound examination due to some inconvenience; (b) incomplete information on medical history; (c) having a history of prostate cancer, bladder cancer, urinary tract stones, or tumors affecting normal urination; and (4) currently taking drugs that affect PV or prostate function.

A structured case report form was designed to collect basic characteristics including age (years), medical history, nation, family history of diseases, allergic history, height (cm), weight (kg), marital status, smoking status, alcohol consumption status, systolic blood pressure (SBP, mmHg), diastolic blood pressure (DBP, mmHg), and fasting blood glucose (ng/ml). BMI was calculated as weight (kg)/height (m^2^).

All participants underwent professional examinations. PV (ml) was measured using B-ultrasonography or magnetic resonance imaging (MRI) for all participants in supine position and then calculated using a standard formula {PV = π/6×[width (cm) × thickness (cm) × length (cm)]} (Terris & Stamey, 1991). IPSS stands for an international and validated tool to measure the severity of LUTS, and the information it required was obtained. IPSS comprises of seven questions and scores from 0 to 35. A score of 0 to 7 indicated mild symptoms, 8 to 19, moderate, and 20 to 35, severe. The urinary flow is calculated through a flow meter, fitted in a urinal, based upon the power necessary to maintain a rotation speed. A graphic printout of the urinary flow is obtained and time taken to reach maximum flow, maximum and average flow rates, and the voided volume are analyzed. The patient performs the test with a comfortably full bladder, voiding volumes of at least 150 ml. *Q*_max_ is an alternative indicator in diagnosing enlarged prostate and was assessed through a noninvasive urodynamic test. *Q*_max_ <10 ml/s represented an abnormal level (Pessoa & Kim, 2018). PVR urine was assessed through transabdominal B-ultrasonography with a PVR threshold at 50 ml, according to the EAU Guidelines.

### Statistical Analysis

Categorical variables were described as frequencies and percentages, and continuous ones as means plus/minus standard deviation, or as medians with interquartile ranges when data were skewed. In accordance with the standards of the National Institutes of Health, the patients were classified into four groups according to BMI: underweight (with BMI values < 18.5 kg/m^2^), normal weight (with BMI values of 18.5–23.0 kg/m^2^), overweight (with BMI values of 23.0–25.0 kg/m^2^), and obese (with BMI values of ≥25.0 kg/m^2^; "Clinical Guidelines on the Identification, Evaluation, and Treatment of Overweight and Obesity in Adults–The Evidence Report. National Institutes of Health," 1998). Clinical cutoffs dividing PV, IPSS, *Q*_max_, and PVR were 80 ml, 19, 10 ml/s, and 50 ml, respectively. Besides, mild and moderate cases were combined in the current study because few patients were at mild level. Univariable logistic regression and multivariable logistic regression was applied to explore the relationship between BMI and dependent variables (categorical PV, IPSS, *Q*_max_, and PVR). In multivariable Model 1, age, nation, smoking status, and alcohol consumption status were adjusted. Model 2 further adjusted SBP, DBP, family history of diseases, allergic history, and fasting blood sugar based on Model 1. Odds ratios (ORs) with 95% confidence intervals (95% CIs) and corresponding *p* value were presented for simplification. A restricted cubic spline (RCS) in logistic regression was carried out to explore potential nonlinear relationship (https://cdn1.sph.harvard.edu/wp-content/uploads/sites/271/2012/09/lgtphcurv9_7-3-2011.pdf). The RCS method was realized adopting SAS macro %lgtphcurv9 and a nice graph was created. Two-side *p* ≤ .05 was considered statistically significant. All analyses were carried out using SAS software, version 9.4 TS1M6 (SAS Institute Inc, Cary, NC).

## Results

A total of 788 BPH patients were included in the current study, and basic characteristics are summarized into Table 1. Their mean age was 72.48 ± 7.32 years, ranging from 52 to 93 years, and BMI ranged from 14.88 to 36.44 kg/m^2^, with a mean value of 23.24 kg/m^2^. Of 788 BPH patients, 745 patients (95.81%) were Chinese Han people, 761 patients (97.07%) reported no family history of diseases, 717 patients (91.22%) reported no allergic history, and only 3 were unmarried. Since all enrolled participants were males, the percentage of smoking and alcohol consumption was high, reaching 34.11% (234 patients) and 25.26% (172 patients), respectively. One hundred and eight patients (13.83%) were diagnosed with hypertension. Fasting blood glucose ranged from 1.016 to 18.45 mmol/L, with a median (Q1, Q3) of 5.10 (4.61, 5.68) mmol/L.

**Table 1.** Baseline Characteristics of the Patients (*n* = 788).

Characteristics	Descriptive statistics
Age (years)	
Mean ± *SD*	72.48 ± 7.32
The Han Chinese (*n* [%])	754 (95.81%)
Smoking status (*n* [%])	
Yes	234 (34.11%)
No	452 (65.89%)
Alcohol consumption status (*n* [%])	
Yes	172 (25.26%)
No	509 (74.74%)
Family history of diseases (*n* [%])	
Yes	23 (2.93%)
No	761 (97.07%)
Marital status (*n* [%])	
Married	783 (99.62%)
Unmarried	3 (0.38%)
Allergic history (*n* [%])	
Yes	69 (8.78%)
No	717 (91.22%)
Body mass index (kg/m^2^)	
Mean ± *SD*	23.24 ± 3.44
Height (cm)	
Mean ± *SD*	168.18 ± 5.67
Weight (kg)	
Mean ± *SD*	65.73 ± 10.39
Systolic blood pressure (mmHg)	
Mean ± *SD*	133.13 ± 17.06
Diastolic blood pressure (mmHg)	
Mean ± *SD*	79.21 ± 10.67
Hypertension status (*n* [%])	
Yes	108 (13.83%)
No	673 (86.17%)
Fasting blood glucose (ng/ml)	
Median (Q1, Q3)	5.10 (4.61, 5.68)

Characteristics of participants’ prostates were presented in Table 2. All participants had nonmissing values for PV, with a median PV of 58.56 ml and ranging from 9.17 ml to 243.8 ml; 226 patients (28.68%) exhibited PV greater than 80 ml. The majority of the patients (758/788) had valid IPSS scores for analysis, with a mean value of 23.92 ± 6.24; only 9 patients (1.19%) presented mild level of IPSS, while 586 (77.31%) had IPSS greater than 19. However, *Q*_max_ and PVR were alternative tools, and about half of the patients underwent both tests. As a result, the median value of *Q*_max_ was 7.3 ml/s, and 281 patients had abnormal *Q*_max_ <10 ml/s; while the median value of PVR was 58 ml, 203 patients had abnormal PVR greater than 50 ml.

**Table 2.** Characteristics of Prostates in Participants.

Characteristics	Descriptive statistics
Prostate volume (PV; ml)	
Median (Q1, Q3)	58.56 (38.73, 85.18)
Nonmissing number (*n* [%])	788
≤80 ml (*n* [%])	562 (71.32%)
>80 ml (*n* [%])	226 (28.68%)
International Prostate Symptom Score (IPSS)	
Mean ± *SD*	23.92 ± 6.24
Nonmissing number (*n* [%])	758
≤19 (*n* [%])	172 (22.69%)
>19 (*n* [%])	586 (77.31%)
0–7 (*n* [%])	9 (1.19%)
8–19 (*n* [%])	163 (21.50%)
>19 (*n* [%])	586 (77.31%)
Maximum urinary flow rate (*Q*_max_; ml/s)	
Median (Q1, Q3)	7.30 (4.60,10.30)
Non-missing number (*n* [%])	385
≤10 ml/s (*n* [%])	281 (72.99%)
>10 ml/s (*n* [%])	104 (27.01%)
Post-void residual (PVR; ml)	
Median (Q1, Q3)	58 (18, 160)
Nonmissing number (*n* [%])	390
≤50 ml (*n* [%])	187 (47.95%)
>50 ml (*n* [%])	203 (52.05%)

Totally, 46 participants were in the underweight group, 298 in normal weight group, 189 in overweight group, and 196 in obese group. Binary logistic regressions were conducted with higher PV, higher IPSS, lower *Q*_max_, and higher PVR as dependent variables and BMI was categorized with the four groups as predictor of interest. In addition, the normal weight group was considered as the reference group. With PV, neither the underweight nor the overweight group presented significant association in univariable regression. There was also no significant association after adjustment in Models 1 and 2. But the obese group faced an increased risk of higher PV in the crude model (OR = 1.772, 95% CI = [1.201, 2.614], *p* = .004). After initial adjustment for age, nation, marriage status, smoking status, and alcohol consumption status, the obese group still showed increased risk of higher PV when compared with the normal group (OR = 1.883, 95% CI [1.226, 2.894], *p* = .004). Further adjustment did not alter the result qualitatively either (OR = 1.912, 95% CI [1.212, 3.017], *p* = .005). In univariable and multivariable regressions, BMI showed no statistically significant effect on higher IPSS, lower *Q*_max_, or higher PVR. Data are summarized into Table 3.

**Table 3.** Risk of Negative Characteristics Associated With BMI Using a Series of Logistic Regressions.

Dependent variable		BMI group (kg/m^2^)						
		Underweight (<18.5 kg/m^2^) *N* = 46		Normal weight (18.5–23.0 kg/m^2^) *N* = 298	Overweight (23.0–25.0 kg/m^2^) *N* = 189		Obese (25.0 kg/m^2^) *N* = 196	
		OR (95% CI)	*p*	OR	OR (95% CI)	*p*	OR (95% CI)	*p*
PV >80 vs. PV ≤80	Crude	0.812 (0.384, 1.714)	.584	1.00 (reference)	1.080 (0.714, 1.632)	.717	1.772 (1.201, 2.614)	.004
	Model 1	0.513 (0.201, 1.309)	.163	1.00 (reference)	0.870 (0.544, 1.391)	.561	1.883 (1.226, 2.894)	.004
	Model 2	0.487 (0.175, 1.353)	.168	1.00 (reference)	0.879 (0.537, 1.438)	.607	1.912 (1.212, 3.017)	.005
IPSS >19 vs. IPSS ≤19	Crude	1.712 (0.691, 4.240)	.245	1.00 (reference)	0.829 (0.532, 1.293)	.408	0.911 (0.585, 1.421)	.682
	Model 1	1.544 (0.608, 3.918)	.361	1.00 (reference)	0.858 (0.542, 1.359)	.515	0.970 (0.612, 1.538)	.898
	Model 2	1.466 (0.568, 3.785)	.429	1.00 (reference)	0.836 (0.520, 1.344)	.459	0.911 (0.566, 1.466)	.701
*Q*_max_ ≤10 vs. *Q*_max_ >10	Crude	0.844 (0.285, 2.495)	.759	1.00 (reference)	0.598 (0.330, 1.082)	.089	0.686 (0.381, 1.233)	.207
	Model 1	1.110 (0.282, 4.368)	.881	1.00 (reference)	0.556 (0.288, 1.072)	.08	0.679 (0.357, 1.291)	.238
	Model 2	0.951(0.233, 3.882)	.945	1.00 (reference)	0.533 (0.271, 1.046)	.067	0.623 (0.319, 1.217)	.166
PVR >50 vs. ≤50	Crude	0.789 (0.295, 2.112)	.638	1.00 (reference)	0.870 (0.520, 1.455)	.595	0.748 (0.452, 1.237)	.258
	Model 1	0.889 (0.314, 2.518)	.825	1.00 (reference)	0.755 (0.435, 1.309)	.317	0.673 (0.394, 1.149)	.147
	Model 2	0.799 (0.273, 2.338)	.682	1.00 (reference)	0.733 (0.414, 1.299)	.288	0.637 (0.366, 1.108)	.11

*Note*. BMI = body mass index; CI = confidence interval; IPSS = International Prostate Symptom Score; OR = odds ratio; PV = prostate volume; PVR = post-void residual; *Q*_max_ = maximum urinary flow rate.

Crude: unadjusted model. Model 1: adjusted for age, nation, smoking status, alcohol consumption status. Model 2: further adjusted for systolic blood pressure, diastolic blood pressure, family history of diseases, allergic history, and fasting blood sugar.

**Figure 1.**
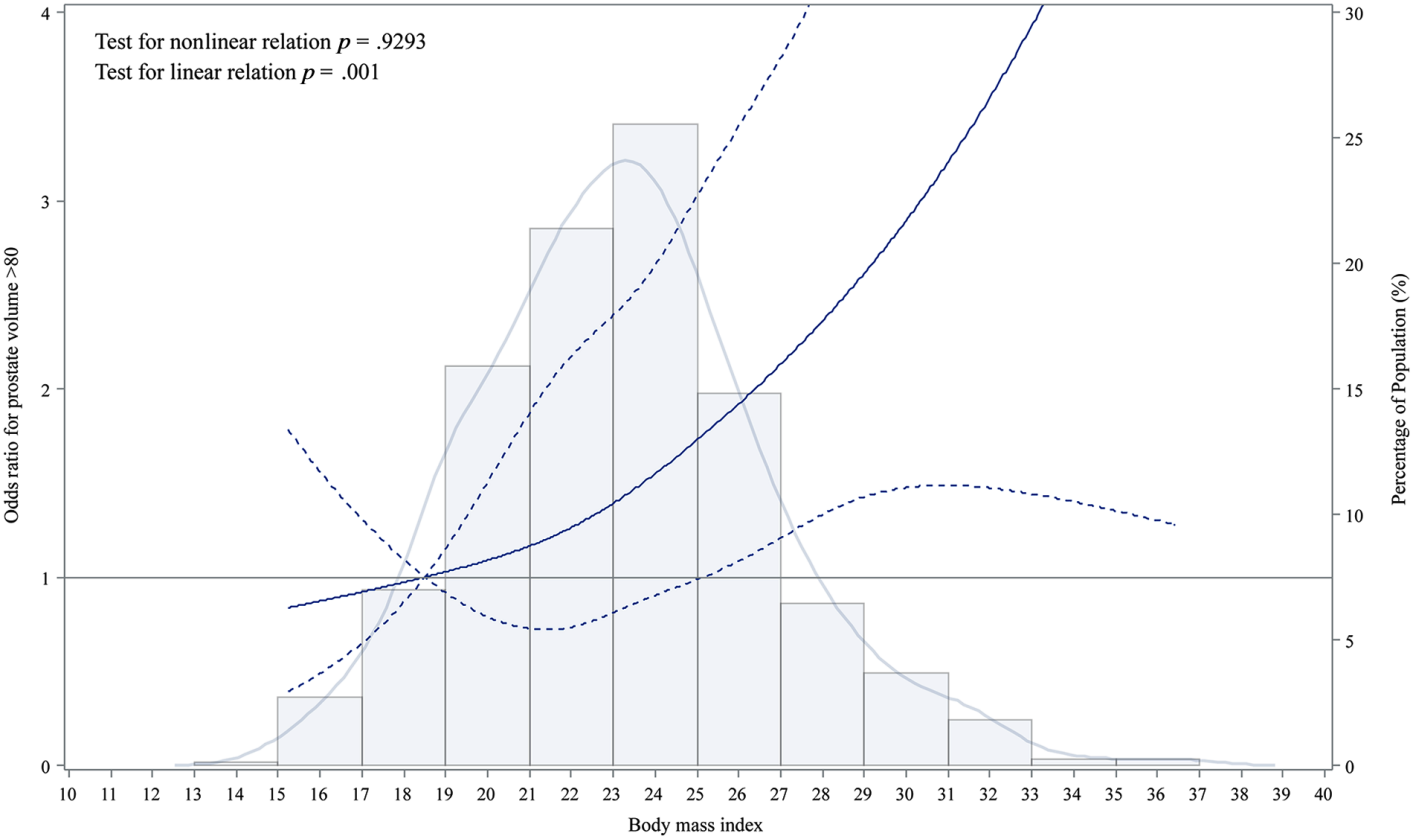
Linear relationship between body mass index and prostate volume through restricted cubic spline method.

In addition, BMI, recorded as a continuous variable, could be explored for its nonlinear relationship with the aforementioned dichotomous dependent variables. An RCS was constructed to present nonlinear relationship. The SAS macro named lgtphcurv9 was employed, in which both nonlinear and linear relationship between BMI and OR were tested simultaneously. Only linear relationship between BMI and OR of higher PV was found (*p* = .001; Figure 1). The relationship was visualized with BMI as X axis and OR for larger PV as Y axis. Moreover, the distribution of BMI was added as background in Figure 1.

## Discussion

Most symptomatic BPH patients need to receive surgical treatment. EAU Guidelines recommend monopolar TURP as a preferred option for patients whose PV is between 30 and 80 cc (Oelke et al., 2013). Moreover, published studies on plasmakinetic energy transurethral resection of the prostate (TUPKP) also have focused on patients with small-to-medium prostate glands (Huang, Li, Yang, Zeng, & Wang, 2015) due to associated complications. Hence, a published study evaluated the efficacy and safety of TUPKP in treating male BPH patients with PV >80 cc (Huang et al., 2015). Although relevant results indicated that TUPKP was safe and efficacious for male BPH cases with large prostate, surgical risk and postoperative complication risk were higher for this group than for patients with small-to-medium prostate. Hence, the exploration of risk factors for PV in BPH patients is an effective approach to prevent surgery-related risks.

Obesity is a medical condition of accumulated excessive body fat and is a known serious health problem in society. The American Medical Association has classified obesity as a disease since 2013, which has been proved to be strongly associated with increasing mortality and morbidity of various disorders, including some cancers, cardiovascular disease, disability, diabetes mellitus, hypertension, osteoarthritis, and stroke (Smith & Smith, 2016). Numerous epidemiological studies have investigated the association between obesity and risk of BPH. For example, three large cohort studies reported that obesity was significantly correlated with the risk of symptomatic BPH (Giovannucci et al., 1994; Jung et al., 2016; Kristal et al., 2007). The first study investigated the frequency and severity of symptoms of urinary obstruction among respondents through questionnaires in 1992 and suggested that abdominal obesity in men might increase both the frequency and severity of urinary obstructive symptoms in Boston patients (Giovannucci et al., 1994). The study by Kristal et al., based on the data from the Prostate Cancer Prevention Trial in the United States, identified that obesity, particularly abdominal obesity, was associated with increased BPH risk (Kristal et al., 2007). Both reported significant increases in obese symptomatic BPH (IPSS >14). Yelsel et al. reported that PV and IPSS values were higher in obese patients than in normal subjects, with statistically significant difference between obese patients and those in other BMI groups (Yelsel et al., 2015). A recent study in Korea also indicated abdominal obesity and serum leptin level were positively associated with prostate growth, whereas serum adiponectin level was negatively associated with the presence of BPH (Jung et al., 2016). Chen et al. reported that IPSS and PV increased with blood glucose levels and BMI in elderly patients with type 2 diabetes. In addition, they also reported that *Q*_max_ decreased with increases in blood glucose levels and BMI (Chen, Miao, Gao, Wang, & Xu, 2015). However, only one study indicated that BMI was associated with PVR in postmenopausal women (Ablove, 2010) and a study detected a negative correlation between ultrasound-estimated bladder weight and PVR (*r* = −0.213, *p* = .033) in men with LUTS (Bright, Pearcy, & Abrams, 2011), while no study has investigated the association between BMI and PVR in BPH patients.

In 2017, a study on Korean men over 80 years investigated the correlation between BMI and routine parameters including PV, in men over 50 years and suggested that PV increased with BMI, which is consistent with the current findings. That study also indicated that despite larger PV in obese individuals, obesity did not aggravate LUTS (Seo et al., 2017). Another study of prostate cancer in northwestern China supported that obesity was inversely correlated with prostate-specific antigen levels in Chinese men and that obese men exhibited significantly larger PV (Zhang, Ma, Nan, & Sheng, 2016). Such a conclusion is consistent with the current findings. However, a major difference existed between these studies and the current study, that is, the current study focused on BPH patients.

To our knowledge, the present study is the first one focusing on the association of BMI with PV, IPSS, *Q*_max_, and PVR in Chinese BPH patients. As a result, this study found a significant linear association between BMI and the risk of larger PV over 80 ml. However, this study did not observe a statistical difference in BMI, IPSS, *Q*_max_, or PVR in patients with BPH. Some studies indicated that total PV growth was associated with aging (Zhang et al., 2016). A considerable proportion of aged men have a stable or decreasing prostate size (Li et al., 2016). In addition, PV growth was also related to socioeconomic status (Rundle, Richards, Neslund-Dudas, Tang, & Rybicki, 2012). Other evidence identified that PV had no correlation with age, symptom score, or QoL score (Agrawal, Chalise, & Bhandari, 2008). The current study also adjusted age, occupation, and marital status. Besides, considering that the transrectal B-ultrasonography was the most frequently recommended technique for examining PV (Terris & Stamey, 1991), the detection technique of B-ultrasonography (transrectal or transabdominal) was also adjusted. Interestingly, the current results revealed nonsignificant association between PV and these factors. Third, current results would have some implications for clinical practice and further research. When performing an operation for BPH patients, the urologist should consider the patients’ BMI and PV rather than only PV, especially the range of obese weight. The mechanisms of why and how only obese weight significantly correlates with PV need further research.

The current study had several limitations. First of all, the present study did not perform analysis on other obesity indices than BMI. IPSS scores were subjective self-assessments from the patients, and possible bias might negatively impact the objectivity in measuring IPSS. Second, investigating the association between other parameters (such as prostate configuration and transition zone volume) and IPSS would be better than investigating that between PV and IPSS; however, owing to a lack of such parameters in our available data, relevant investigations were performed. Hence, this aspect probably should be considered in future studies. Third, in an observational study, different designs hold varied evidence levels, prospective cohort design possessing the highest level, followed by case–control design and cross-sectional design. The current study adopted a cross-sectional design, so the level of evidence and persuasive power were both limited. Therefore, prospective cohort studies would be able in future to obtain stronger evidence on this topic. At least, a case–control study with healthy controls is workable.

In conclusion, the present cross-sectional study uncovered significant linear association between BMI and the risk of PV over 80 ml. BMI is significantly related to PV, and obese weight would significantly increase PV. These results recommend that the urologist should consider both BMI and PV when planning surgical treatment for BPH patients.
